# Thyroid stimulating immunoglobulin concentration is associated with disease activity and predicts response to treatment with intravenous methylprednisolone in patients with Graves’ orbitopathy

**DOI:** 10.3389/fendo.2024.1340415

**Published:** 2024-03-21

**Authors:** Gijsbert J. Hötte, P. Martijn Kolijn, Maaike de Bie, Ronald O. B. de Keizer, Marco Medici, Kim van der Weerd, P. Martin van Hagen, Dion Paridaens, Willem A. Dik

**Affiliations:** ^1^ Department of Oculoplastic, Lacrimal & Orbital Surgery, Rotterdam Eye Hospital, Rotterdam, Netherlands; ^2^ Laboratory Medical Immunology, Department of Immunology, Erasmus MC University Medical Center Rotterdam, Rotterdam, Netherlands; ^3^ Academic Center for Thyroid Diseases, Department of Internal Medicine, Erasmus MC University Medical Center Rotterdam, Rotterdam, Netherlands; ^4^ Department of Internal Medicine, Franciscus Gasthuis & Vlietland, Rotterdam, Netherlands; ^5^ Department of Internal Medicine, Section Clinical Immunology, Erasmus MC University Medical Center Rotterdam, Rotterdam, Netherlands; ^6^ Department of Ophthalmology, Erasmus MC University Medical Center Rotterdam, Rotterdam, Netherlands

**Keywords:** graves’ orbitopathy, disease activity, treatment response, methylprednisolone, TSI, sIL-2R

## Abstract

**Background:**

Thyroid stimulating immunoglobulins (TSI) play a central role in the pathogenesis of Graves’ orbitopathy (GO), while soluble interleukin-2 receptor (sIL-2R) is a marker for T-cell activity. We investigated TSI and sIL-2R levels in relation to thyroid function, disease activity and severity and response to treatment with intravenous methylprednisolone (IVMP) in patients with GO.

**Methods:**

TSI (bridge-based TSI binding assay), sIL-2R, TSH and fT4 levels were measured in biobank serum samples from 111 GO patients (37 male, 74 female; mean age 49.2 years old) and 25 healthy controls (5 male, 20 female; mean age 39.8 years old). Clinical characteristics and response to treatment were retrospectively retrieved from patient files.

**Results:**

Higher sIL-2R levels were observed in GO patients compared to controls (p < 0.001). sIL-2R correlated with fT4 (*r* = 0.26), TSH (*r* = -0.40) and TSI (*r* = 0.21). TSI and sIL-2R concentrations were higher in patients with active compared to inactive GO (p < 0.001 and p < 0.05, respectively). Both TSI and sIL-2R correlated with total clinical activity score (CAS; *r* = 0.33 and *r* = 0.28, respectively) and with several individual CAS items. Cut-off levels for predicting active GO were 2.62 IU/L for TSI (AUC = 0.71, sensitivity 69%, specificity 69%) and 428 IU/mL for sIL-2R (AUC = 0.64, sensitivity 62%, specificity 62%). In multivariate testing higher TSI (p < 0.01), higher age (p < 0.001) and longer disease duration (p < 0.01) were associated with disease activity. TSI levels were higher in patients with a poor IVMP response (p = 0.048), while sIL-2R levels did not differ between responders and non-responders. TSI cut-off for predicting IVMP response was 19.4 IU/L (AUC = 0.69, sensitivity 50%, specificity 91%). In multivariate analysis TSI was the only independent predictor of response to IVMP (p < 0.05).

**Conclusions:**

High TSI levels are associated with active disease (cut-off 2.62 IU/L) and predict poor response to IVMP treatment (cut-off 19.4 IU/L) in GO. While sIL-2R correlates with disease activity, it is also related to thyroid function, making it less useful as an additional biomarker in GO.

## Introduction

Graves’ orbitopathy (GO) is an autoimmune condition characterized by inflammation and volume expansion of the soft tissues that surround the eye, resulting in proptosis, eyelid retraction, edema, restricted ocular motility and diplopia (1). It arises from a complex cellular interplay involving T-cells, B-cells, mast cells and orbital fibroblasts (OF) (2, 3). Orbital fibroblasts express the thyroid-stimulating hormone (TSH) receptor (TSH-R) and activation of the OF is, among other stimuli, triggered by binding of TSH-R autoantibodies (TSH-R-Ab or TRAb) (4, 5).

TSH-R-Ab can be further divided into stimulating autoantibodies (TSAb, also referred to as TSH-R stimulating immunoglobulins [TSI]), blocking autoantibodies (TBAb, also known as TSH-R blocking immunoglobulins [TBI]), or neutral autoantibodies that do not interfere with TSH binding to the receptor (5, 6). Competitive-binding immunoassays that detect the total of stimulatory and blocking TSH-R-Ab, also referred to as TSH-R-binding inhibitory immunoglobulins (TBII), are commonly used in clinical practice (5). Although these TBII assays display good sensitivity and specificity, they do not provide information on the actual functionality of the antibodies (i.e. stimulating or blocking) (5). Selective detection of autoantibodies with either TSH-R stimulating or blocking properties is possible with technically more challenging functional cell-based bioassays (5–7). More recently, an automated bridge-based binding assay has become commercially available (Immulite^®^ 2000 TSI immunoassay) (8, 9). This assay uses a TSH-R chimera and has been designed to detect TSI more specifically, although not exclusively since a number of studies show that certain TBI are also detected (10–12).

Because of its central role in the pathogenesis, the use of TSH-R-Ab as a biomarker for GO has been investigated. Research herein has primarily focused on the use of different assays as a biomarker for disease activity and severity, showing that TSI bioassays, outperform TBII assays (4, 5, 7, 13–25). So far, only a limited number of studies investigated the applicability of the bridge-based TSI binding assay as a biomarker for disease activity and severity in GO, with conflicting results (26–30). Although identifying patients with active disease is important, it may be even more relevant to identify patients who will not respond to intravenous methylprednisolone (IVMP). This treatment is still considered the first-line treatment during the phase of active inflammation, but a large portion of patients respond insufficiently (31, 32). Unfortunately, reliable biomarkers to identify these non-responders before IVMP initiation are lacking and so far studies that explored the relation between TSH-R-Ab assays and IVMP response are scarce (26, 33–35).

While research strongly focused on the use of TSH-R-Ab as biomarker for GO, soluble interleukin-2 receptor (sIL-2R) is a biomarker for T-cell activity that is used to evaluate disease activity and treatment response in a variety of immune-mediated diseases, including autoimmune diseases (36, 37). Higher levels of sIL-2R have also been described in patients with Graves’ disease (GD), which correlated with thyroid function (38–43). Interestingly, sIL-2R levels were found to be higher in GD patients with orbitopathy as compared to GD patients without orbitopathy, which may reflect more profound activation of the immune system in the first group (44, 45). However, only few studies have explored the clinical relevance of sIL-2R as a biomarker for GO activity and severity (42, 43, 46, 47) and none have tested the relation between sIL-2R levels and response to IVMP.

The goal of the present study was to investigate serum TSI levels measured with the bridge-based TSI binding assay (Immulite^®^ 2000 TSI immunoassay) and serum sIL-2R levels in relation to thyroid function, disease activity, disease severity, and IVMP treatment response in patients with GO.

## Materials and methods

### Patients and controls

For this study, serum samples that were stored at -80°C in the Combined Ophthalmic Research Rotterdam Biobank (CORRBI) were used. Ethical approval for CORRBI in general was granted by the local medical ethical committee (MEC-2012-031). Informed consent was obtained for all CORRBI participants after being informed on the ethical issues regarding storage and use of samples (48). The use of samples for our study was approved by the biobank committee. All files from patients whose samples were stored under the (tentative) diagnosis of GO were selected for further review. Clinical characteristics, laboratory tests and orbital imaging were evaluated to confirm diagnosis of GO, ultimately resulting in 111 patients that were included. In addition, serum samples from a cohort of 25 healthy individuals were obtained as a control group, as approved by the local medical ethical committee (MEC-2021-0251).

### Clinical evaluation

Medical history and demographic features were recorded for all patients and healthy controls. For GO patients, results from ophthalmological and orbital examination were retrospectively obtained from the patient files. Severity of the condition was determined using the EUGOGO classification (mild, moderate-to-severe, and sight-threatening GO) (31). Disease activity was assessed using the clinical activity score (CAS) of 7 items: spontaneous retrobulbar pain, gaze evoked pain, eyelid erythema, conjunctival hyperemia, eyelid swelling, chemosis and inflammation of the caruncle/plica (49). Active disease was defined as a total CAS of ≥ 3 points in one or both eyes. Patients who were treated with IVMP after the biobank sample was obtained, were evaluated for treatment response. IVMP dosing schemes were based on EUGOGO guidelines and tailored in selected cases depending on comorbidity and side effects (31). For severe disease, the standard scheme included 1000mg of IVMP for three consecutive days, which was repeated if indicated. For moderate-to-severe disease, the standard dosing regimen consisted of a cumulative dose of 4500mg of IVMP in 12 weekly infusions. As part of a recent study by our group, a small subset of patients with moderate-to-severe disease was treated with a regimen of prednisolone-encapsulated liposomes (two times 150mg intravenously with a 2-week interval), that is potentially associated with a more specific targeted delivery of the drug at the inflamed areas and requires fewer hospital visits while typical steroid-related adverse events are reduced (50). A beneficial response to IVMP treatment was defined as (1) achievement of a total CAS < 3 in both eyes, or (2) an improvement of ≥ 2 points in one eye without concomitant deterioration in the fellow eye.

### Serologic evaluation

Serum samples were defrosted and analyzed for free thyroxine (fT4), TSH, TSI and sIL-2R. The analyses were performed under strict quality rules (ISO15189) by the clinical chemistry facility and medical immunology laboratory at Erasmus MC. Reference range was 14 - 29 pmol/L for fT4 and 0.56 - 4.27 mIU/L for TSH. Based on fT4 and TSH results, thyroid function was further classified as hyperthyroid, subclinical hyperthyroid, hypothyroid, subclinical hypothyroid and euthyroid. TSI was measured with Immulite^®^ 2000 TSI immunoassay (Siemens Healthineers AG, Erlangen, Germany) and sIL-2R was measured with Immulite^®^ 2000 IL2R immunoassay. For TSI a cut-off value for positivity of ≥ 0.55 IU/L, as defined by the manufacturer, was used. Our institution’s current cut-off value for elevated sIL-2R is 555 IU/ml.

### Statistical analyses

*Castor EDC* was used as a clinical data management system (51). Data were subsequently exported to *SPSS* v.28 (IBM corp., Armonk, New York, USA) and R Statistical Software (v4.2.2, R Core Team 2021) (52) for statistical analysis. Differences in continuous variables between groups were evaluated using an independent sample t-test or Mann-Whitney U test. For categorical variables Fisher’s exact test was used. Spearman rank correlation coefficient was used for correlation analyses. Receiver operator curve (ROC) analysis was performed using the pROC R package (v1.18.0) (53). Cut-off values based on Youden’s indices were used for dichotomous distribution and subsequently applied to a multivariate logistic regression model.

## Results

### Patient characteristics

**Table 1** summarizes the demographic and clinical data of GO patients (n =111) and healthy controls (n = 25). The clinical data of the GO patients correspond to the visit at which the biobank sample was obtained. In total, 39 patients were treated with IVMP. Of these, 11 patients were treated for severe disease with daily high doses of IVMP (median cumulative dose of 3000mg; IQR = 1000). Furthermore, 24 patients with moderate-to-severe disease were treated with weekly infusions of IVMP, with a median cumulative dose of 4500mg (IQR = 0). Another four patients with moderate-to-severe disease from this cohort received treatment with prednisolone-encapsulated liposomes, with a cumulative dose of 300mg (50). Median time between obtaining the serum sample and the start of IVMP treatment was 11 days (IQR = 39.50). The median duration between completion of IVMP treatment and subsequent clinical evaluation was 16.5 days (IQR 38.50).

**Table 1:** Demographic and clinical data of GO patients and healthy controls.

		Graves’ orbitopathy	Healthy Controls	p-value
		(n = 111)	(n = 25)	
**Sex**	Male	37 (33.3%)	5 (20%)	0.236^a^
	Female	74 (66.7%)	20 (80%)	
**Age (years)**	Mean	49.2 (± 15.7)	39.8 (± 11.5)	< 0.01^b^
**Smoking status**	Smoker	34 (30.6%)	0 (0%)	< 0.001^a^
	Non-smoker	57 (51.4%)	25 (100%)	
	Unknown	20 (18.0%)	0 (0%)	
**Thyroid disease history**	Hyperthyroidism	103 (92.8%)	N/A	N/A
	Hypothyroidism	8 (7.2%)		
**Previous thyroid treatment**	Complete or partial thyroidectomy	11 (9.9%)	N/A	N/A
	Radioactive iodine	25 (22.5%)		
**Current thyroid medication**	Block & replace	35 (31.5%)	N/A	N/A
	Titration	16 (14.4%)		
	Thyroid hormone	37 (33.3%)		
	None	23 (20.7%)		
**Duration of symptoms (months)**	Median	10 (IQR 22)	N/A	N/A
**CAS**	Median	2.0 (IQR 3)	N/A	N/A
	Active (CAS ≥ 3)	45 (40.5%)		
	Inactive (CAS < 3)	65 (58.6%)		
	Unknown	1 (0.9%)		
**Severity**	Mild	14 (12.6%)	N/A	N/A
	Moderate-to-severe	87 (78.4%)		
	Severe	10 (9.0%)		
**Response to IVMP**	Responder	22 (56.4%)	N/A	N/A
	Non-responder	16 (41.0%)		
	Unknown	1 (2.6%)		

CAS (Clinical Activity Score); IVMP (intravenous methylprednisolone); IQR (interquartile range); N/A (not applicable).

a: Fisher’s exact test, Freeman-Halton extension when appropriate.

b: Independent sample t-test.

### Serologic results

Serologic results from patients and controls are shown in **Table 2**. As expected, median TSI was significantly higher in patients compared to controls (p < 0.001) and TSI levels above the reference range were found in 97 GO patients (87.4%) but in none of the healthy controls. Interestingly, GO patients also showed significantly higher median sIL-2R concentrations than the control group (p < 0.001; **Table 2**; **Supplementary Figure 1**). sIL-2R levels above the reference range were observed in 26 patients (23.4%) but not in any of the controls.

**Table 2:** Serologic results in GO patients and healthy controls.

		Graves’ orbitopathy	Healthy controls	p-value
**Current thyroid status**	Euthyroidism	44 (39.6%)	20 (80%)	< 0.001^a^
	Subclinical hypothyroidism	13 (11.7%)	4 (16%)	
	Overt hypothyroidism	11 (9.9%)	1 (4%)	
	Subclinical hyperthyroidism	28 (25.2%)	0 (0%)	
	Overt hyperthyroidism	15 (13.5%)	0 (0%)	
**fT4**	Median	20.7 (IQR 8.5)	18.4 (IQR 3.82)	< 0.05^b^
**(normal range 14 - 29 pmol/L)**	Below normal limits	11 (9.9%)	1 (4%)	0.064^a^
	Within normal limits	85 (76.6%)	24 (96%)	
	Above normal limits	15 (13.5%)	0 (0%)	
**TSH**	Median	1.12 (IQR 3.0)	1.84 (IQR 1.16)	< 0.05^b^
**(normal range 0.56 - 4.27 mIU/L)**	Below normal limits	44 (39.6%)	0 (0%)	<0.001^a^
	Within normal limits	46 (41.4%)	21 (84%)	
	Above normal limits	21 (18.9%)	4 (16%)	
**TSI**	Median	3.36 (IQR 8.29)	<0.10 (IQR 0.0)	< 0.001^b^
**(normal range < 0.55 IU/L)**	Within normal limits	14 (12.6%)	25 (100%)	< 0.001^a^
	Above normal limits	97 (87.4%)	0 (0%)	
**sIL-2R^c^**	Median	418 (IQR 225)	286 (IQR 149)	< 0.001^b^
**(normal range < 555 IU/mL)**	Within normal limits	85 (76.6%)	25 (100%)	< 0.01^a^
	Above normal limits	26 (23.4%)	0 (0%)	

fT4 (free T4, thyroxine); sIL-2R (soluble interleukin-2 receptor); TSH (thyroid-stimulating hormone); TSI (thyroid-stimulating immunoglobulins).

a: Fisher’s exact test, Freeman-Halton extension when appropriate.

b: Mann-Whitney U test.

c: see also **Supplementary Figure 3**.

### Relation between sIL-2R levels and TSI and thyroid function

**Figure 1:**
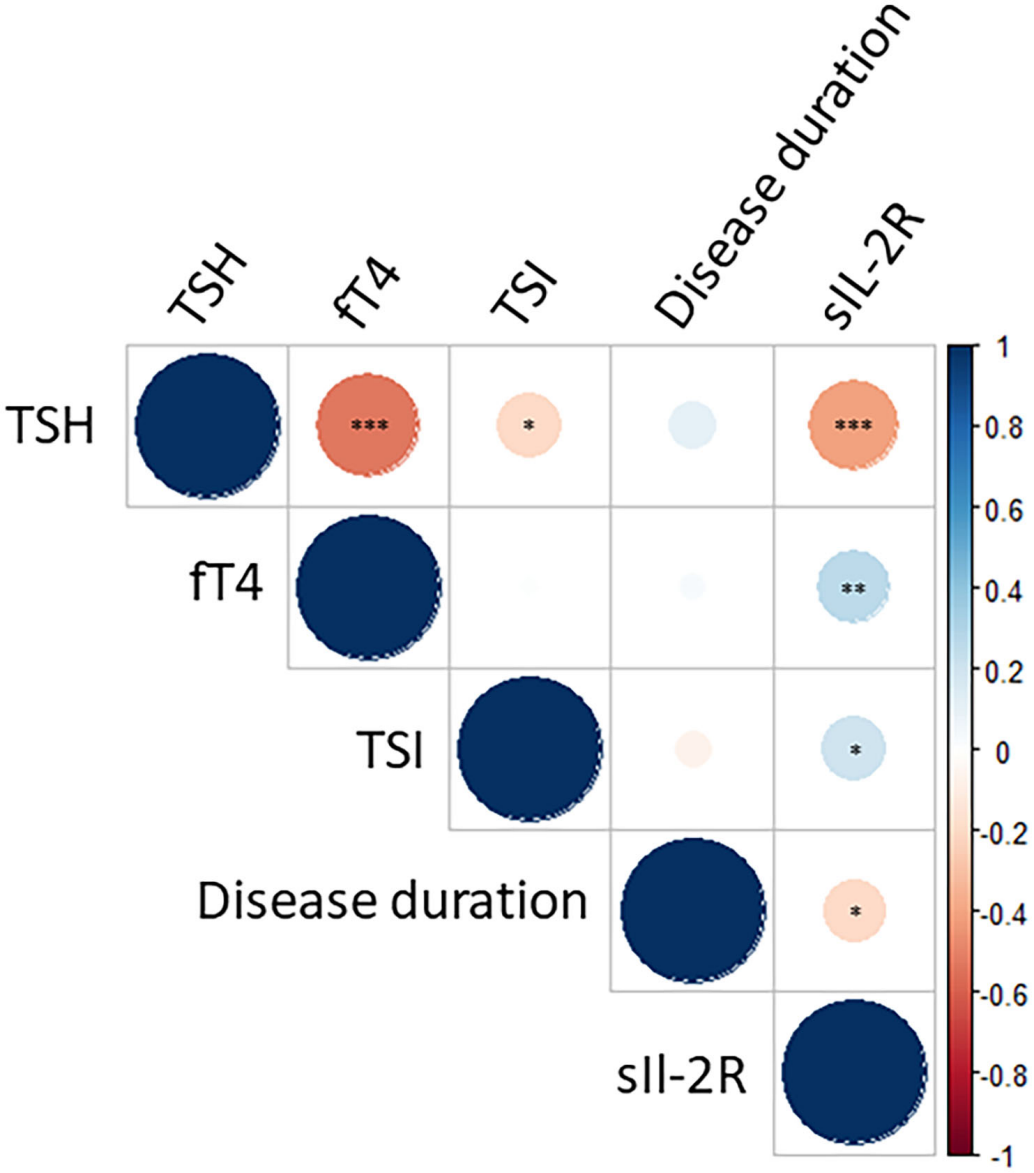
Correlation matrix for sIL-2R, TSI and thyroid function. Dots with a red hue depict a negative correlation, whereas dots with a blue hue represent a positive correlation. Level of significance is depicted with asterisks (* p < 0.05; ** p < 0.01; *** p < 0.001). A significant and positive correlation is found between sIL-2R levels and fT4 (r = 0.26) and TSI levels (r = 0.21), whereas a negative correlation is observed between sIL-2R and TSH (r = -0.40) and duration of symptoms (r = -0.19). For a complete representation, the figure also shows the correlation between TSI and TSH/fT4. However, this correlation is less relevant because in a large proportion of patients thyroid function is regulated by medication.

In patients with GO, we observed a positive correlation between sIL-2R levels and fT4 (*r*= 0.26) and TSI levels (*r*= 0.21). A negative correlation was found between sIL-2R and TSH (*r*= -0.40) and duration of symptoms (*r* = -0.19) (**Figure 1**).

### Relation between TSI and sIL-2R levels and disease severity

TSI and sIL-2R concentrations did not differ between severity groups (mild, moderate-to-severe and severe), although a trend towards lower sIL-2R levels was observed in moderate-to-severe compared to severe GO (**Supplementary Figure 2**).

### Relation between TSI and sIL-2R levels and disease activity

**Figure 2:**
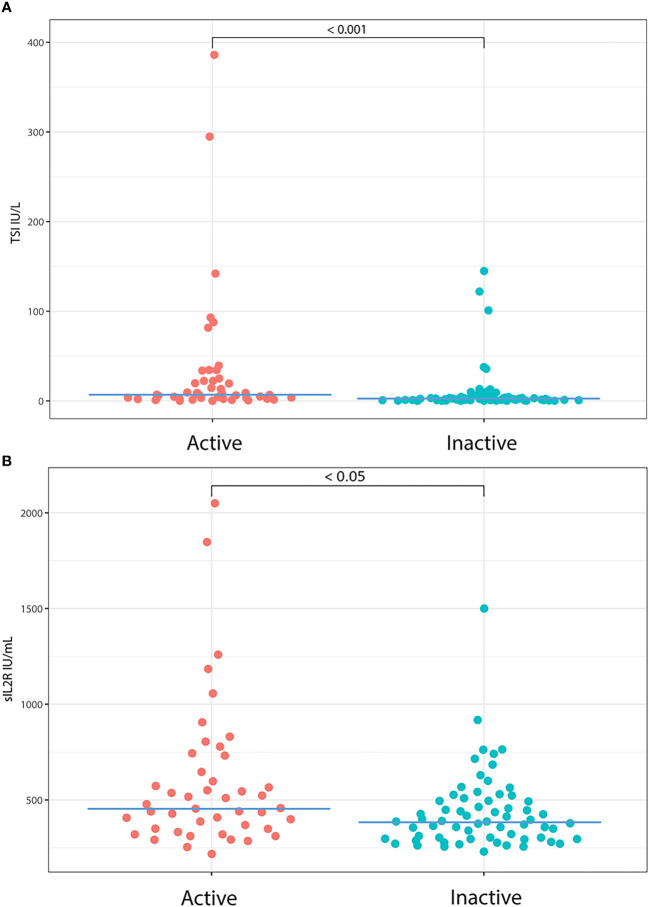
TSI and sIL-2R levels in patients with active and inactive disease. We observed increased TSI and sIL-2R values for a subset of patients with active disease compared to inactive disease. **(A)** Median TSI was 6.80 IU/L (IQR = 21.14) in active GO and 2.53 IU/L (IQR = 4.53) in inactive GO (p < 0.001). **(B)** Median sIL-2R was 457 IU/mL (IQR = 340) in patients with active orbitopathy compared to 387 IU/mL (IQR = 202) in patients with quiescent disease (p < 0.05).

As for disease activity, we observed increased TSI concentrations for a subset of patients with active disease compared to those with quiescent disease (**Figure 2A**). Similar results were found for sIL-2R, showing higher levels in active GO (**Figure 2B**).

**Figure 3:**
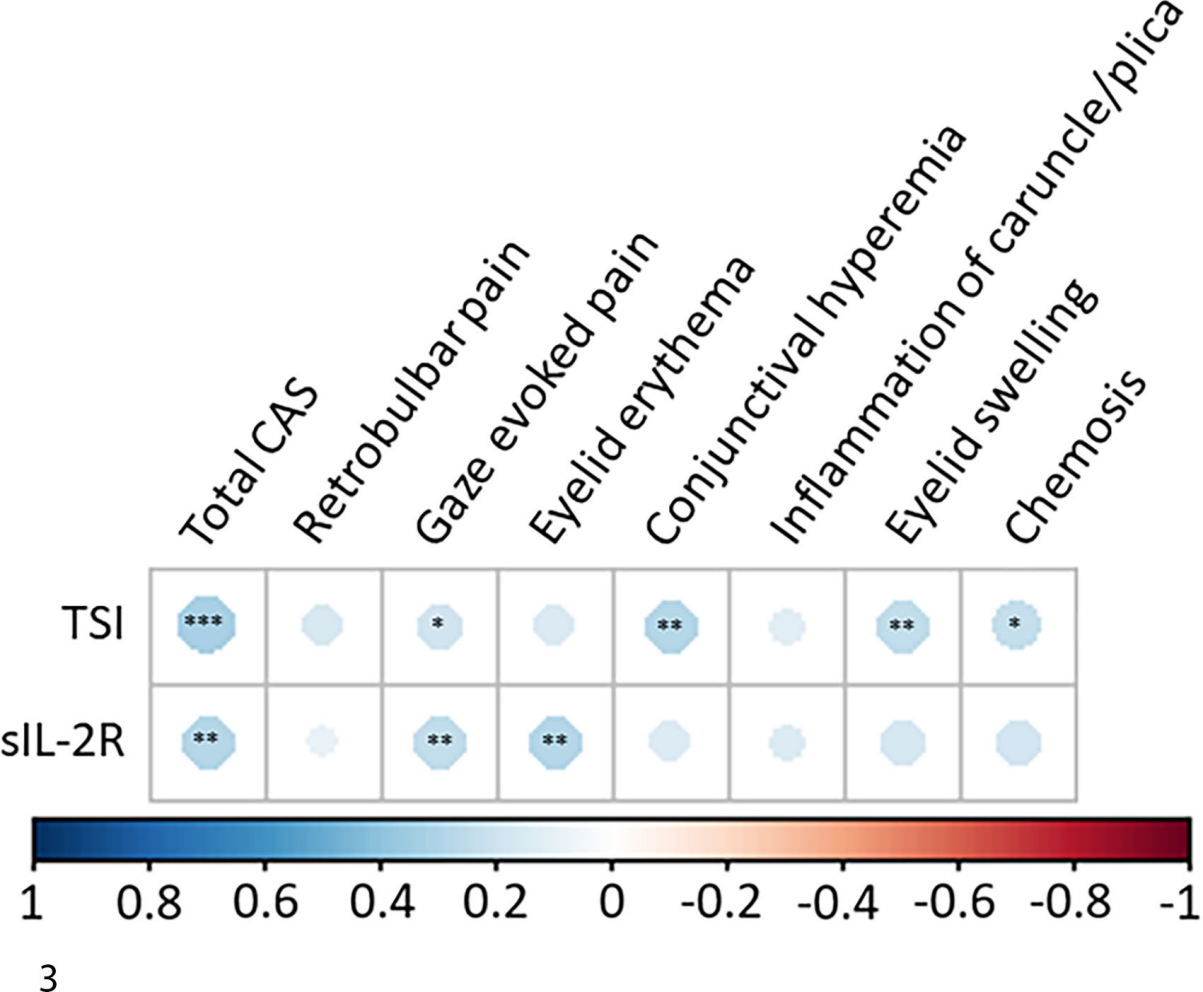
Correlation matrix for sIL-2R and TSI levels and clinical activity score (CAS). Dots with a blue hue depict a positive correlation. Level of significance is depicted with asterisks (* p < 0.05; ** p < 0.01; *** p < 0.001). TSI levels correlated with total CAS (r = 0.33) and with the individual items of gaze evoked pain (r = 0.20), conjunctival hyperemia (r = 0.28), eyelid swelling (r = 0.25) and chemosis (r = 0.23). sIL-2R levels correlated significantly with the total CAS (r = 0.28) and with the individual items of gaze evoked pain (r = 0.25) and eyelid erythema (r = 0.29).

Moreover, a weak correlation was observed for TSI levels with total CAS (*r* = 0.33) and with the individual items of gaze evoked pain (*r* = 0.20), conjunctival hyperemia (*r* = 0.28), eyelid swelling (*r* = 0.25) and chemosis (*r* = 0.23) (**Figure 3**). sIL-2R also displayed a weak correlation with the total CAS (*r* = 0.28) and with the individual items of gaze evoked pain (*r* = 0.25) and eyelid erythema (*r* = 0.29) (**Figure 3**).

**Figure 4:**
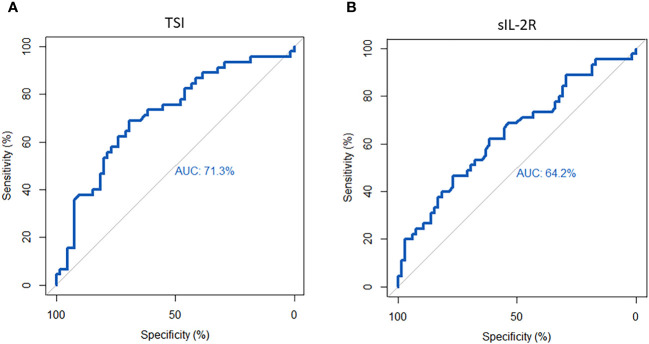
Receiver operator curve (ROC) for sIL-2R and TSI in identifying active disease. **(A)** For TSI, ROC analysis showed an area under the curve (AUC) of 0.71. The cut-off value of 2.62 IU/L represented a 69% sensitivity and 69% specificity. **(B)** For sIL-2R, the AUC was 0.64 and a cut-off value of 428 IU/mL showed a sensitivity of 62% and a specificity of 62%.

For TSI, ROC analysis showed an area under the curve (AUC) of 0.71 for identifying active disease. With Youden’s index, a TSI cut-off value of 2.62 IU/L was associated with a sensitivity of 69% and specificity of 69% (**Figure 4A**). For sIL-2R, the AUC for identifying active disease was 0.64 and a cut-off value of 428 IU/mL displayed a 62% sensitivity and 62% specificity (**Figure 4B**). The cut-off values for TSI and sIL-2R were used for dichotomous distribution (i.e. low or high levels). With these cut-off values, multivariate logistic regression analysis showed that higher TSI, older age and longer disease duration were independently associated with active disease, while sIL-2R was not (**Table 3**).

**Table 3:** Multivariate logistic regression analysis for disease activity.

	OR	CI (95%)	p-value
(Intercept)	0.0062	0.00024 - 0.094	< 0.001
sIL-2R elevated ^a^	2.38	0.68 - 8.82	0.18
**TSI elevated ^b^**	**9.28**	**2.20 - 52.7**	**< 0.01**
**Age**	**1.10**	**1.05 - 1.16**	**< 0.001**
Sex (male)	0.68	0.14 - 3.07	0.61
smoking (unknown)	0.67	0.099 - 3.93	0.66
Smoking (yes)	1.14	0.26 - 5.14	0.86
Hyperthyroidism	1.05	0.12 - 9.95	0.96
Hypothyroidism	2.46	0.27 - 22.6	0.42
Subclinical hyperthyroidism	1.35	0.29 - 6.50	0.70
Subclinical hypothyroidism	3.96	0.42 - 37.9	0.22
**Disease duration**	**0.996**	**0.993 - 0.999**	**< 0.01**

a: Elevated sIL-2R is based on Youden’s index (> 428 IU/mL).

b: Elevated TSI is based on Youden’s index (> 2.62 IU/L).

Bold refers to statistically significant results.

### Relation between TSI and sIL-2R levels and response to treatment with intravenous methylprednisolone

Median TSI levels at baseline were significantly lower in patients that responded to IVMP treatment (3.36 IU/L; IQR = 7.78) compared to non-responders (14.2 IU/L; IQR = 30.46; p = 0.049; **Supplementary Figure 3A**). The difference between these groups remained statistically significant when the four patients treated with prednisolone-encapsulated liposomes were omitted from the analysis (p = 0.046). When evaluating only patients with moderate-to-severe disease, the analyses lost statistical significance. No difference was observed in sIL-2R levels between IVMP responders and non-responders (**Supplementary Figure 3B**). No difference in baseline CAS was found between responders (median 4; IQR 2) and non-responders (median 5; IQR 2; p = 0.43).

**Figure 5:**
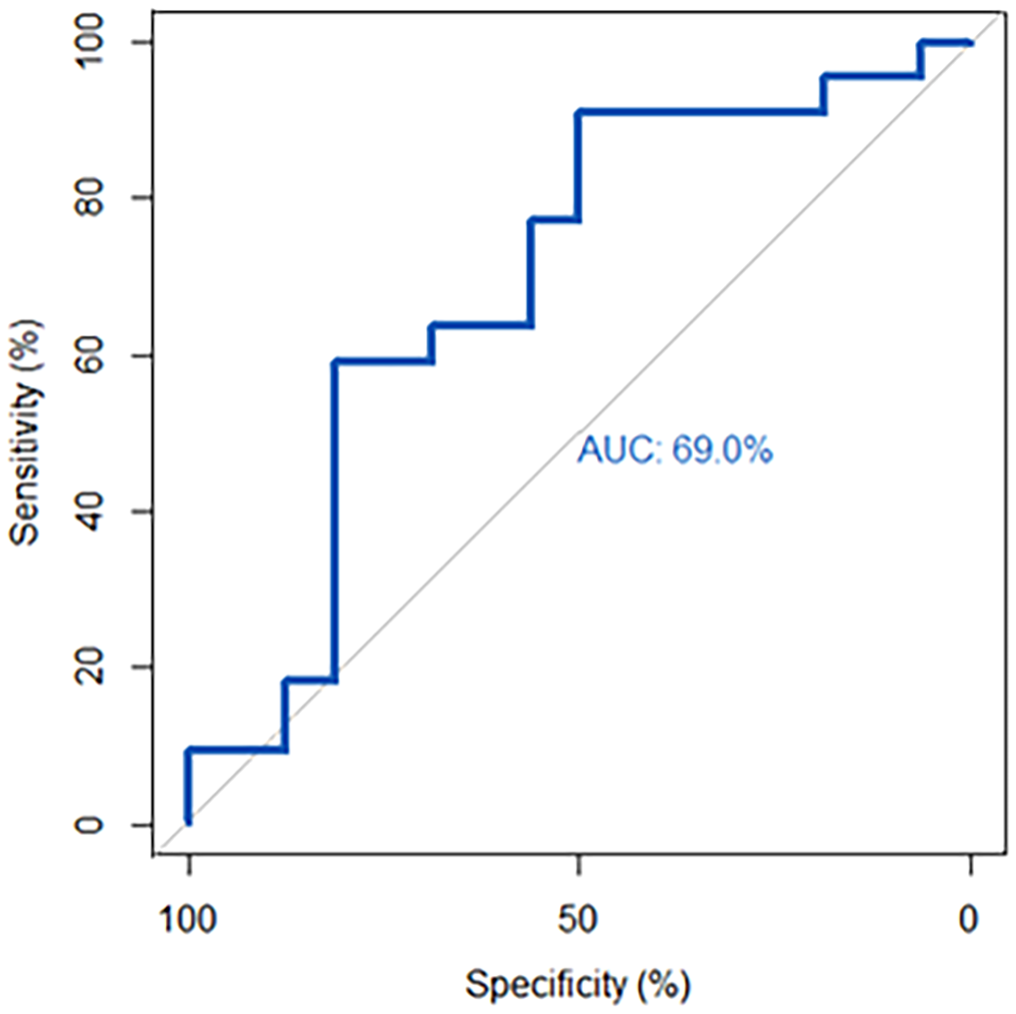
Receiver operator curve (ROC) for TSI in identifying responders to IVMP. ROC-analysis showed an area under the curve (AUC) of 0.69. The cut-off of 19.4 IU/L was associated with a sensitivity of 50% and a specificity of 91%.

ROC analysis for TSI as a marker to identify patients responding to IVMP showed an AUC of 0.69. A cut-off value of 19.4 IU/L was associated with a sensitivity of 50% and a specificity of 91% (**Figure 5**). Based on this cut-off, TSI was the only independent predictor of response to IVMP treatment in multivariate logistic regression (**Table 4**).

**Table 4:** Multivariate logistic regression analysis for response to treatment with methylprednisolone.

	OR	CI (95%)	p-value
(Intercept)	6.31	0.16 - 442.7	0.34
**TSI (low) ^a^**	**13.8**	**1. 97 - 171.2**	**< 0.05**
Age	0.95	0.89 - 1.01	0.099
Sex (male)	0.68	0.098 - 4.33	0.68
Smoking (unknown)	3.33	0.18 - 136.8	0.45
Smoking (yes)	0.23	0.031 - 1.35	0.12

a: low TSI is based on Youden’s index (< 19.4 IU/L).

Bold refers to statistically significant results.

## Discussion

This is the first study to demonstrate that high TSI levels (measured with the bridge-based TSI binding assay: Immulite^®^ 2000 TSI immunoassay) are associated with poor response to IVMP treatment in patients with GO, as observed in both univariate and multivariate analyses. The calculated TSI cut-off value of 19.4 IU/L for predicting a favorable treatment response showed low sensitivity (50%) but high specificity (91%), indicating half of responders could be accurately identified, with limited false positives among non-responders. These findings suggest that switching to alternative immunosuppressive treatment at an earlier stage, or starting additional immunosuppression simultaneously with IVMP, may be beneficial in patients with high TSI levels. Also, while antithyroid drug treatment and thyroidectomy are generally associated with a reduction of TSH-R-Ab levels, radioactive iodine (RAI) may lead to an increase (54). Therefore, our study provides additional support for the consensus that RAI should be avoided in patients with active orbitopathy, especially in those requiring IVMP treatment. Interestingly, the only other study that investigated the relation between the bridge-based TSI binding assay and IVMP response could not identify such a difference (26). However, in their study patients who experienced an improvement in CAS of ≥ 1 were considered responders, which may explain the discrepancies in outcome. As for other types of antibody assays, a limited number of studies have explored the potential of TSH-R-Ab to predict IVMP response. Studies by Leo et al. and De Bellis et al. did not identify TBII as an independent predictor for treatment response (33, 34). On the other hand, Park et al. demonstrated that higher TSI bioassay levels were in fact linked to a poor response to IVMP, although with low magnitude (OR 1.005; p = 0.038) (35).

The mechanisms through which high TSI levels result in poor response to IVMP are currently unclear. Presumably, high TSI levels are associated with a more profound disease process and overactive inflammatory state. In this biological context, the suppressive signal exerted by IVMP may potentially be less effective. This assumption is strengthened by our observation that high TSI levels are also associated with disease activity in both univariate and multivariate analysis. However, it appears to be in contrast to a study by Mourits et al. in which an association between high pre-treatment CAS and a favorable response to oral prednisolone, was observed (55). This association could not be confirmed in our study and this discrepancy may be explained by the fact that they also included patients with pre-treatment CAS < 3. Although they used the NOSPECS score to define treatment response, it has many similarities with the CAS in terms of soft tissue signs. With both scoring systems it is perhaps more likely that an improvement is obtained when, for example, the initial CAS is 7. After all, there will be 7 items available for improvement. This is in contrast to when, for example, the CAS is 2, which puts only 2 items available for improvement, while 5 others are eligible to deterioration.

The degree of correlation that we observed between TSI and total CAS was weak and the cut-off value of 2.62 IU/L for active disease showed limited discriminative value. These results are inferior to those from studies that determined true functional biological activity of TSH-R-Ab with specific bioassays (5, 18), possibly because the bridge-based binding assay used in our study still detects certain TBAb/TBI (10–12). Interestingly, although the bridge-based assay presumably detects TSI more specifically than TBII assays, studies found that TBII assays correlate equally or even stronger with disease activity than the bridge-based assay (26, 30). Furthermore, our study did not find any correlation between bridge-based TSI levels and the severity of the disease, which is consistent with some previous reports (26, 28), but differing from another (27).

Although the AUC that we found for TSI in predicting activity and IVMP response is regarded to be sufficient (56), it is desirable to identify additional biomarkers associated with disease activity and IVMP response. Because T-cells contribute to GO pathogenesis, we hypothesized that sIL-2R, which is shed by activated T-cells, could serve as another biomarker in GO (36, 37). Consistent with other studies, we demonstrated that sIL-2R is elevated in GO patients compared to healthy controls and that serum levels correlate with thyroid function (38–43). Furthermore, we observed higher sIL-2R concentrations in patients with active GO compared to inactive disease, which is in line with results from Mariotti et al., but in contrast to a study by Wakelkamp et al. (42, 43) Comparable to TSI, serum sIL-2R also showed a weak correlation with total CAS. However, after correcting for thyroid function, sIL-2R was not independently associated with disease activity. These results support previous reports indicating that elevated sIL-2R levels in GD are related to thyroid function per se, rather than the underlying autoimmune process (38, 41). High levels of sIL-2R in the context of hyperthyroidism can be explained by the interplay between thyroid hormones and T-cells. Thyroid hormones are known to cause increased numbers of activated T-cells in GD patients, which normalize after restoration of thyroid function (41, 57–60). Because of the relation with thyroid function, sIL-2R is not a valuable biomarker for immune activity in GO. Additionally, we found no statistically significant association between sIL-2R and disease severity, which is consistent with other studies, although a trend towards lower sIL-2R levels was observed in moderate-to-severe compared to severe GO (p = 0.059) (43, 46).

Furthermore, we could not identify a relation between sIL-2R serum levels and response to IVMP. While our study is the first to investigate such an association, a prior study by Prummel et al. found that GO patients with elevated sIL-2R tended to have greater probability to respond to oral prednisone, although this did not reach statistical significance (p = 0.081) (46).

Interestingly, although both serum TSI and sIL-2R showed a weak correlation with total CAS, the levels did not correlate separately with all individual items of the CAS, which is consistent with the findings of a recently published study that was conducted at the same time as ours (61). However, as it is unlikely that a single biomarker would be able to capture all facets of the disease process, the lack of correlation between some of the CAS items and TSI or sIL-2R does not necessarily imply that these items have no clinical value. As such, our data do not suffice to present a proposal regarding a modified CAS. Nonetheless, these results, together with known limitations of the current CAS, do suggest that it is valuable to further investigate whether all items of the CAS have equal clinical and biological relevance, and whether some elements could be replaced or supplemented with biomarkers (31, 49, 62).

Our study has several limitations. sIL-2R concentrations increase with age and in our study GO patients were significantly older than the healthy controls (49.2 vs. 39.8 years old, respectively) (63). However, because the age effect is seen particularly after 65 years, we consider it unlikely that this has had a major impact on our observations (63). Studies have also shown that sIL-2R is higher in smokers and this may also have affected the comparison between GO patients and healthy (non-smoking) controls (63). Nonetheless, we did not find a difference in sIL-2R levels when we compared smokers and non-smokers within our GO group (443.5 IU/mL, IQR = 200 vs. 428 IU/mL, IQR = 247; p = 0.68).

A further consideration is the heterogeneity of our study population. The dosing scheme of IVMP varied depending on the specific indication (severe or moderate-to-severe) and our cohort also included four patients who, as part of a trial, were treated with prednisolone-encapsulated liposomes. The differences in TSI levels between responders and non-responders remained statistically significant when these four patients were removed from the analysis. However, when evaluating patients with moderate-to-severe disease only, significance was lost, which may be due to the low number of patients.

While the CAS is the most commonly used and best validated scoring system for disease activity and response to treatment, it has several limitations (31). Although we were unable to do so in our current retrospective study, we plan to validate our findings in future studies using other modalities to grade disease activity, such as magnetic resonance imaging (MRI).

Moreover, in this retrospective study TSI and sIL-2R levels were only measured at a single point during the disease course. Although disease duration was added in the multivariate model, it would be ideal to include longitudinal serologic data. By doing so, serum levels can be correlated to disease activity along the course of Rundle’s curve more precisely.

A final point of attention is that a relation between serum levels and disease severity in our study may have been missed because of the small numbers of patients with mild or severe disease. While severe disease is rather rare in general, the number of mild cases in our cohort is limited because the study reflects a tertiary referral center.

In summary, high TSI levels measured with the bridge-based binding assay are independently associated with active disease (cut-off 2.62 IU/L) and predict poor response to treatment with IVMP (cut-off 19.4 IU/L) in GO patients. However, because of the limited AUC for TSI in predicting activity and IVMP response, it is desirable to identify additional biomarkers for this clinically important application. While sIL-2R correlates with disease activity in univariate analysis, it is also related to thyroid function, making it less useful as an additional biomarker in GO. A prospective study is currently underway validating the results of our current study and exploring the applicability of other biomarkers.

## Data availability statement

The datasets presented in this article are not readily available because na. Requests to access the datasets should be directed to g.hotte@oogziekenhuis.nl.

## Ethics statement

The studies involving humans (biobank and healthy controls) were approved by the local medical ethical committe of the Erasmus MC (MEC-2012-031 and MEC-2021-0251). The studies were conducted in accordance with the local legislation and institutional requirements. The participants provided their written informed consent to participate in this study.

## Author contributions

GH: Writing – original draft, Formal analysis, Data curation. PK: Writing – review & editing, Visualization, Formal analysis. MB: Writing – review & editing, Investigation. RD: Writing – review & editing, Funding acquisition. MM: Writing – review & editing. KV: Writing – review & editing. PV: Writing – review & editing, Supervision. DP: Writing – review & editing, Supervision, Conceptualization. WD: Writing – review & editing, Supervision, Conceptualization.

## Supplementary material

Supplementary Figure 1 sIL-2R levels in GO patients and healthy controls. sIL-2R levels are significantly higher in GO patients (418 IU/mL, IQR = 225) compared to healthy controls (286 IU/mL, IQR = 149; p < 0.001).

Supplementary Figure 2 TSI and sIL-2R levels among severity groups. **(A)** Median TSI levels were 1.92 IU/L (IQR = 13.21) in mild disease, 3.36 IU/L (IQR = 8.27) in moderate-to-severe and 4.27 IU/L (IQR = 13.6) in severe GO. The differences were not statistically significant. **(B)** Median sIL-2R concentrations were 392 IU/mL (IQR = 245) in mild GO, 409 IU/mL (IQR = 219) in moderate-to-severe and 543.5 IU/mL (IQR = 236) in severe disease. The trend towards lower sIL-2R levels in moderate-to-severe compared to severe GO did not reach statistical significance (p = 0.059).

Supplementary Figure 3 TSI and sIL-2R levels in patients responders and non-responders to IVM. **(A)** Higher TSI levels were observed in non-responders (14.2 IU/L; IQR = 30.46) compared to responders (3.36 IU/L; IQR = 7.78 p = 0.049). **(B)** No difference in sIL-2R levels was found between responders (459.5 IU/mL; IQR = 261) and non-responders (474.5 IU/mL, IQR = 394; p = 0.35).

## References

[1] Bahn RS. Graves' ophthalmopathy. N Engl J Med. (2010) 362:726–38. doi: 10.1056/NEJMra0905750 PMC3902010 20181974

[2] Lee ACH Kahaly GJ. Pathophysiology of thyroid-associated orbitopathy. Best Pract Res Clin Endocrinol Metab. (2023) 37:101620. doi: 10.1016/j.beem.2022.101620 35181241

[3] Dik WA Virakul S van Steensel L. Current perspectives on the role of orbital fibroblasts in the pathogenesis of Graves' ophthalmopathy. Exp Eye Res. (2016) 142:83–91. doi: 10.1016/j.exer.2015.02.007 26675405

[4] Diana T Kahaly GJ. Thyroid stimulating hormone receptor antibodies in thyroid eye disease-methodology and clinical applications. Ophthalmic Plast Reconstr Surg. (2018) 34:S13–S9. doi: 10.1097/IOP.0000000000001053 29771755

[5] Diana T Ponto KA Kahaly GJ. Thyrotropin receptor antibodies and Graves' orbitopathy. J Endocrinol Invest. (2021) 44:703–12. doi: 10.1007/s40618-020-01380-9 PMC8310479 32749654

[6] Kahaly GJ Diana T. TSH receptor antibody functionality and nomenclature. Front Endocrinol (Lausanne). (2017) 8:28. doi: 10.3389/fendo.2017.00028 28261158 PMC5309226

[7] Lytton SD Ponto KA Kanitz M Matheis N Kohn LD Kahaly GJ. A novel thyroid stimulating immunoglobulin bioassay is a functional indicator of activity and severity of Graves' orbitopathy. J Clin Endocrinol Metab. (2010) 95:2123–31. doi: 10.1210/jc.2009-2470 20237164

[8] Frank CU Braeth S Dietrich JW Wanjura D Loos U. Bridge technology with TSH receptor chimera for sensitive direct detection of TSH receptor antibodies causing graves' Disease: analytical and clinical evaluation. Horm Metab Res. (2015) 47:880–8. doi: 10.1055/s-0035-1554662 26079838

[9] Tozzoli R D'Aurizio F Villalta D Giovanella L. Evaluation of the first fully automated immunoassay method for the measurement of stimulating TSH receptor autoantibodies in Graves' disease. Clin Chem Lab Med. (2017) 55:58–64. doi: 10.1515/cclm-2016-0197 27331310

[10] Diana T Wuster C Kanitz M Kahaly GJ. Highly variable sensitivity of five binding and two bio-assays for TSH-receptor antibodies. J Endocrinol Invest. (2016) 39:1159–65. doi: 10.1007/s40618-016-0478-9 27197966

[11] Diana T Wuster C Olivo PD Unterrainer A König J Kanitz M. Performance and specificity of 6 immunoassays for TSH receptor antibodies: A multicenter study. Eur Thyroid J. (2017) 6:243–9. doi: 10.1159/000478522 PMC5649260 29071236

[12] Allelein S Diana T Ehlers M Kanitz M Hermsen D Schott M. Comparison of a bridge immunoassay with two bioassays for thyrotropin receptor antibody detection and differentiation. Horm Metab Res. (2019) 51:341–6. doi: 10.1055/a-0914-0535 31207654

[13] Dragan LR Seiff SR Lee DC. Longitudinal correlation of thyroid-stimulating immunoglobulin with clinical activity of disease in thyroid-associated orbitopathy. Ophthalmic Plast Reconstr Surg. (2006) 22:13–9. doi: 10.1097/01.iop.0000192649.23508.f7 16418659

[14] George A Diana T Langericht J Kahaly GJ. Stimulatory thyrotropin receptor antibodies are a biomarker for graves' Orbitopathy. Front Endocrinol (Lausanne). (2020) 11:629925. doi: 10.3389/fendo.2020.629925 33603715 PMC7885640

[15] Gerding MN van der Meer JW Broenink M Bakker O Wiersinga WM Prummel MF. Association of thyrotrophin receptor antibodies with the clinical features of Graves' ophthalmopathy. Clin Endocrinol (Oxf). (2000) 52:267–71. doi: 10.1046/j.1365-2265.2000.00959.x 10718823

[16] Goh SY Ho SC Seah LL Fong KS Khoo DH. Thyroid autoantibody profiles in ophthalmic dominant and thyroid dominant Graves' disease differ and suggest ophthalmopathy is a multiantigenic disease. Clin Endocrinol (Oxf). (2004) 60:600–7. doi: 10.1111/j.1365-2265.2004.02033.x 15104563

[17] Jang SY Shin DY Lee EJ Choi YJ Lee SY Yoon JS. Correlation between TSH receptor antibody assays and clinical manifestations of Graves' orbitopathy. Yonsei Med J. (2013) 54:1033–9. doi: 10.3349/ymj.2013.54.4.1033 PMC3663223 23709442

[18] Jeon H Lee JY Kim YJ Lee MJ. Clinical relevance of thyroid-stimulating immunoglobulin as a biomarker of the activity of thyroid eye disease. Eye (Lond). (2023) 37:543–7. doi: 10.1038/s41433-022-01981-z PMC9905053 35220401

[19] Kahaly GJ Diana T Kanitz M Frommer L Olivo PD. Prospective trial of functional thyrotropin receptor antibodies in graves disease. J Clin Endocrinol Metab. (2020) 105:e1006–14. doi: 10.1210/clinem/dgz292 PMC7067543 31865369

[20] Kampmann E Diana T Kanitz M Hoppe D Kahaly GJ. Thyroid stimulating but not blocking autoantibodies are highly prevalent in severe and active thyroid-associated orbitopathy: A prospective study. Int J Endocrinol. (2015) 2015:678194. doi: 10.1155/2015/678194 26221139 PMC4499387

[21] Khoo DH Ho SC Seah LL Fong KS Tai ES Chee SP. The combination of absent thyroid peroxidase antibodies and high thyroid-stimulating immunoglobulin levels in Graves' disease identifies a group at markedly increased risk of ophthalmopathy. Thyroid. (1999) 9:1175–80. doi: 10.1089/thy.1999.9.1175 10646655

[22] Noh JY Hamada N Inoue Y Abe Y Ito K Ito K. Thyroid-stimulating antibody is related to Graves' ophthalmopathy, but thyrotropin-binding inhibitor immunoglobulin is related to hyperthyroidism in patients with Graves' disease. Thyroid. (2000) 10:809–13. doi: 10.1089/thy.2000.10.809 11041459

[23] Ponto KA Diana T Binder H Matheis N Pitz S Pfeiffer N. Thyroid-stimulating immunoglobulins indicate the onset of dysthyroid optic neuropathy. J Endocrinol Invest. (2015) 38:769–77. doi: 10.1007/s40618-015-0254-2 25736545

[24] Ponto KA Kanitz M Olivo PD Pitz S Pfeiffer N Kahaly GJ. Clinical relevance of thyroid-stimulating immunoglobulins in graves' ophthalmopathy. Ophthalmology. (2011) 118:2279–85. doi: 10.1016/j.ophtha.2011.03.030 21684605

[25] Poon SHL Cheung JJ Shih KC Chan YK. A systematic review of multimodal clinical biomarkers in the management of thyroid eye disease. Rev Endocr Metab Disord. (2022) 23:541–67. doi: 10.1007/s11154-021-09702-9 35066781

[26] Bluszcz GA Bednarczuk T Bartoszewicz Z Kondracka A Walczak K Żurecka Z. Clinical utility of TSH receptor antibody levels in Graves' orbitopathy: a comparison of two TSH receptor antibody immunoassays. Cent Eur J Immunol. (2018) 43:405–12. doi: 10.5114/ceji.2018.80224 PMC6384424 30799988

[27] Stohr M Oeverhaus M Lytton SD Horstmann M Zwanziger D Möller L. Predicting the course of graves' Orbitopathy using serially measured TSH-receptor autoantibodies by automated binding immunoassays and the functional bioassay. Horm Metab Res. (2021) 53:435–43. doi: 10.1055/a-1525-2070 34282595

[28] Thia B McGuinness MB Ebeling PR Khong JJ. Diagnostic accuracy of Immulite(R) TSI immunoassay for thyroid-associated orbitopathy in patients with recently diagnosed Graves' hyperthyroidism. Int Ophthalmol. (2022) 42:863–70. doi: 10.1007/s10792-021-02052-0 34613563

[29] Khamisi S Lundqvist M Engstrom BE Larsson A Karlsson FA Ljunggren Ö. Comparison between thyroid stimulating immunoglobulin and TSH-receptor antibodies in the management of graves' Orbitopathy. Exp Clin Endocrinol Diabetes. (2023) 131:236–41. doi: 10.1055/a-2021-0596 PMC10158629 36706788

[30] Moledina M Roos J Murthy R. Thyrotropin receptor autoantibody assessment in thyroid eye disease: does the assay type matter? Korean J Ophthalmol. (2023) 37:147–56. doi: 10.3341/kjo.2022.0131 PMC10151161 37080243

[31] Bartalena L Kahaly GJ Baldeschi L Dayan CM Eckstein A Marcocci C. The 2021 European Group on Graves' orbitopathy (EUGOGO) clinical practice guidelines for the medical management of Graves' orbitopathy. Eur J Endocrinol. (2021) 185:G43–67. doi: 10.1530/EJE-21-0479 34297684

[32] Bartalena L Krassas GE Wiersinga W Marcocci C Salvi M Daumerie C. Efficacy and safety of three different cumulative doses of intravenous methylprednisolone for moderate to severe and active Graves' orbitopathy. J Clin Endocrinol Metab. (2012) 97:4454–63. doi: 10.1210/jc.2012-2389 23038682

[33] De Bellis A Bizzarro A Conte M Coronella C Solimeno S Perrino S. Relationship between longitudinal behaviour of some markers of eye autoimmunity and changes in ocular findings in patients with Graves' ophthalmopathy receiving corticosteroid therapy. Clin Endocrinol (Oxf). (2003) 59:388–95. doi: 10.1046/j.1365-2265.2003.01861.x 12919164

[34] Leo M Mautone T Ionni I Profilo MA Sabini E Menconi F. Variables affecting the long-term outcome of graves orbitopathy following high-dose intravenous glucocorticoid pulse therapy in patients not treated with orbital radiotherapy. Endocr Pract. (2016) 22:1177–86. doi: 10.4158/E161376.OR 27732097

[35] Park J Kim J Ryu D Choi HY. Factors related to steroid treatment responsiveness in thyroid eye disease patients and application of SHAP for feature analysis with XGBoost. Front Endocrinol (Lausanne). (2023) 14:1079628. doi: 10.3389/fendo.2023.1079628 36817584 PMC9928572

[36] Damoiseaux J. The IL-2 - IL-2 receptor pathway in health and disease: The role of the soluble IL-2 receptor. Clin Immunol. (2020) 218:108515. doi: 10.1016/j.clim.2020.108515 32619646

[37] Dik WA Heron M. Clinical significance of soluble interleukin-2 receptor measurement in immune-mediated diseases. Neth J Med. (2020) 78:220–31. 33093245

[38] Jiskra J Antosova M Limanova Z Telicka Z Lacinová Z. The relationship between thyroid function, serum monokine induced by interferon gamma and soluble interleukin-2 receptor in thyroid autoimmune diseases. Clin Exp Immunol. (2009) 156:211–6. doi: 10.1111/j.1365-2249.2009.03897.x PMC2759467 19250272

[39] Pedro AB Romaldini JH Takei K. Changes of serum cytokines in hyperthyroid Graves' disease patients at diagnosis and during methimazole treatment. Neuroimmunomodulation. (2011) 18:45–51. doi: 10.1159/000311519 20628263

[40] Zwirska-Korczala K Berdowska A Jochem J Sitkiewicz A Birkner E Polaniak R. Influence of thyroxine on serum soluble interleukin-2 receptor alpha levels in thyroid disorders. J Clin Pharm Ther. (2004) 29:151–6. doi: 10.1111/j.1365-2710.2004.00547.x 15068404

[41] Koukkou E Panayiotidis P Alevizou-Terzaki V Thalassinos N. High levels of serum soluble interleukin-2 receptors in hyperthyroid patients: correlation with serum thyroid hormones and independence from the etiology of the hyperthyroidism. J Clin Endocrinol Metab. (1991) 73:771–6. doi: 10.1210/jcem-73-4-771 1909702

[42] Mariotti S Caturegli P Barbesino G Marinò M Del Prete GF Chiovato L. Thyroid function and thyroid autoimmunity independently modulate serum concentration of soluble interleukin 2 (IL-2) receptor (sIL-2R) in thyroid diseases. Clin Endocrinol (Oxf). (1992) 37:415–22. doi: 10.1111/j.1365-2265.1992.tb02352.x 1486691

[43] Wakelkamp IM Gerding MN van der Meer JW Prummel MF Wiersinga WM. Both Th1- and Th2-derived cytokines in serum are elevated in Graves' ophthalmopathy. Clin Exp Immunol. (2000) 121:453–7. doi: 10.1046/j.1365-2249.2000.01335.x PMC1905733 10971510

[44] Balazs C Farid NR. Soluble interleukin-2 receptor in sera of patients with Graves' disease. J Autoimmun. (1991) 4:681–8. doi: 10.1016/0896-8411(91)90185-F 1777014

[45] Balazs CS. Increased level of soluble interleukin-2 receptor in sera of patients with Graves' disease. BioMed Pharmacother. (1991) 45:311–4. doi: 10.1016/0753-3322(91)90086-9 1760523

[46] Prummel MF Wiersinga WM van der Gaag R Mourits MP Koornneef L. Soluble IL-2 receptor levels in patients with Graves' ophthalmopathy. Clin Exp Immunol. (1992) 88:405–9. doi: 10.1111/j.1365-2249.1992.tb06462.x PMC1554494 1606722

[47] Terwee CB Prummel MF Gerding MN Kahaly GJ Dekker FW Wiersinga WM. Measuring disease activity to predict therapeutic outcome in Graves' ophthalmopathy. Clin Endocrinol (Oxf). (2005) 62:145–55. doi: 10.1111/j.1365-2265.2005.02186.x 15670189

[48] Meester M Vingerling JR Dorrestijn N Dorrestijn N Klaver CCW Ingeborgh Van Den Born L. Professional storage of clinical biosamples for eye diseases: the CORRBI biobank. Invest Ophthalmol Visual Science. (2015) 56:1382–.

[49] Mourits MP Koornneef L Wiersinga WM Prummel MF Berghout A van der Gaag R. Clinical criteria for the assessment of disease activity in Graves' ophthalmopathy: a novel approach. Br J Ophthalmol. (1989) 73:639–44. doi: 10.1136/bjo.73.8.639 PMC1041835 2765444

[50] Detiger SE Kremer TM Dalm VASH de Keizer ROB Wubbels ROB Metselaar RJ Metselaar JM. A pilot study on the use of prednisolone-encapsulated liposomes for the treatment of moderate-to-severe Graves' orbitopathy with reduced systemic steroid exposure. Acta Ophthalmol. (2021) 99:797–804. doi: 10.1111/aos.14751 33423386

[51] Castor EDC. Castor Electronic Data Capture. (2019). Available online at: https://castoredc.com.

[52] R Core Team. R: A language and environment for statistical computing. Vienna, Austria: R Foundation for Statistical Computing (2021). Available at: https://www.R-project.org/.

[53] Robin X Turck N Hainard A Tiberti N Lisacek F Sanchez JC. pROC: an open-source package for R and S+ to analyze and compare ROC curves. BMC Bioinf. (2011) 12:77. doi: 10.1186/1471-2105-12-77 PMC3068975 21414208

[54] Bartalena L Piantanida E Gallo D Lai A Tanda ML. Epidemiology, natural history, risk factors, and prevention of graves' Orbitopathy. Front Endocrinol (Lausanne). (2020) 11:615993. doi: 10.3389/fendo.2020.615993 33329408 PMC7734282

[55] Mourits MP Prummel MF Wiersinga WM Koornneef L. Clinical activity score as a guide in the management of patients with Graves' ophthalmopathy. Clin Endocrinol (Oxf). (1997) 47:9–14. doi: 10.1046/j.1365-2265.1997.2331047.x 9302365

[56] Simundic AM. Measures of diagnostic accuracy: basic definitions. EJIFCC. (2009) 19:203–11. PMC4975285 27683318

[57] Wenzek C Boelen A Westendorf AM Engel DR Moeller LC Führer D. The interplay of thyroid hormones and the immune system - where we stand and why we need to know about it. Eur J Endocrinol. (2022) 186:R65–77. doi: 10.1530/EJE-21-1171 PMC9010816 35175936

[58] van der Weerd K van Hagen PM Schrijver B Heuvelmans SJ Hofland LJ Swagemakers SM. Thyrotropin acts as a T-cell developmental factor in mice and humans. Thyroid. (2014) 24:1051–61. doi: 10.1089/thy.2013.0396 24635198

[59] Van der Weerd K Van Hagen PM Schrijver B Kwekkeboom DJ De Herder WW Ten Broek MR. The peripheral blood compartment in patients with Graves' disease: activated T lymphocytes and increased transitional and pre-naive mature B lymphocytes. Clin Exp Immunol. (2013) 174:256–64. doi: 10.1111/cei.12183 PMC3828829 23901889

[60] Ishikawa N Eguchi K Otsubo T Ueki Y Fukuda T Tezuka H. Reduction in the suppressor-inducer T cell subset and increase in the helper T cell subset in thyroid tissue from patients with Graves' disease. J Clin Endocrinol Metab. (1987) 65:17–23. doi: 10.1210/jcem-65-1-17 2953751

[61] Saric Matutinovic M Kahaly GJ Zarkovic M Ćirić J Ignjatović S Nedeljković Beleslin B. The phenotype of Graves' orbitopathy is associated with thyrotropin receptor antibody levels. J Endocrinol Invest. (2023) 46:2309–17. doi: 10.1007/s40618-023-02085-5 37020104

[62] Dickinson AJ Perros P. Controversies in the clinical evaluation of active thyroid-associated orbitopathy: use of a detailed protocol with comparative photographs for objective assessment. Clin Endocrinol (Oxf). (2001) 55:283–303. doi: 10.1046/j.1365-2265.2001.01349.x 11589671

[63] Alende-Castro V Alonso-Sampedro M Fernández-Merino C Sopeña B Vidal C Gude F. Factors influencing serum concentrations of soluble interleukin-2 receptor: a general adult population study. All Life. (2023) 16. doi: 10.1080/26895293.2023.2169958

