# Supplementary figures and images for: Thyroid stimulating immunoglobulin concentration is associated with disease activity and predicts response to treatment with intravenous methylprednisolone in patients with Graves’ orbitopathy
Source: Front Endocrinol (Lausanne). 2024 Mar 21;15:1340415. doi: 10.3389/fendo.2024.1340415 (PMC10993908; doi:10.3389/fendo.2024.1340415)

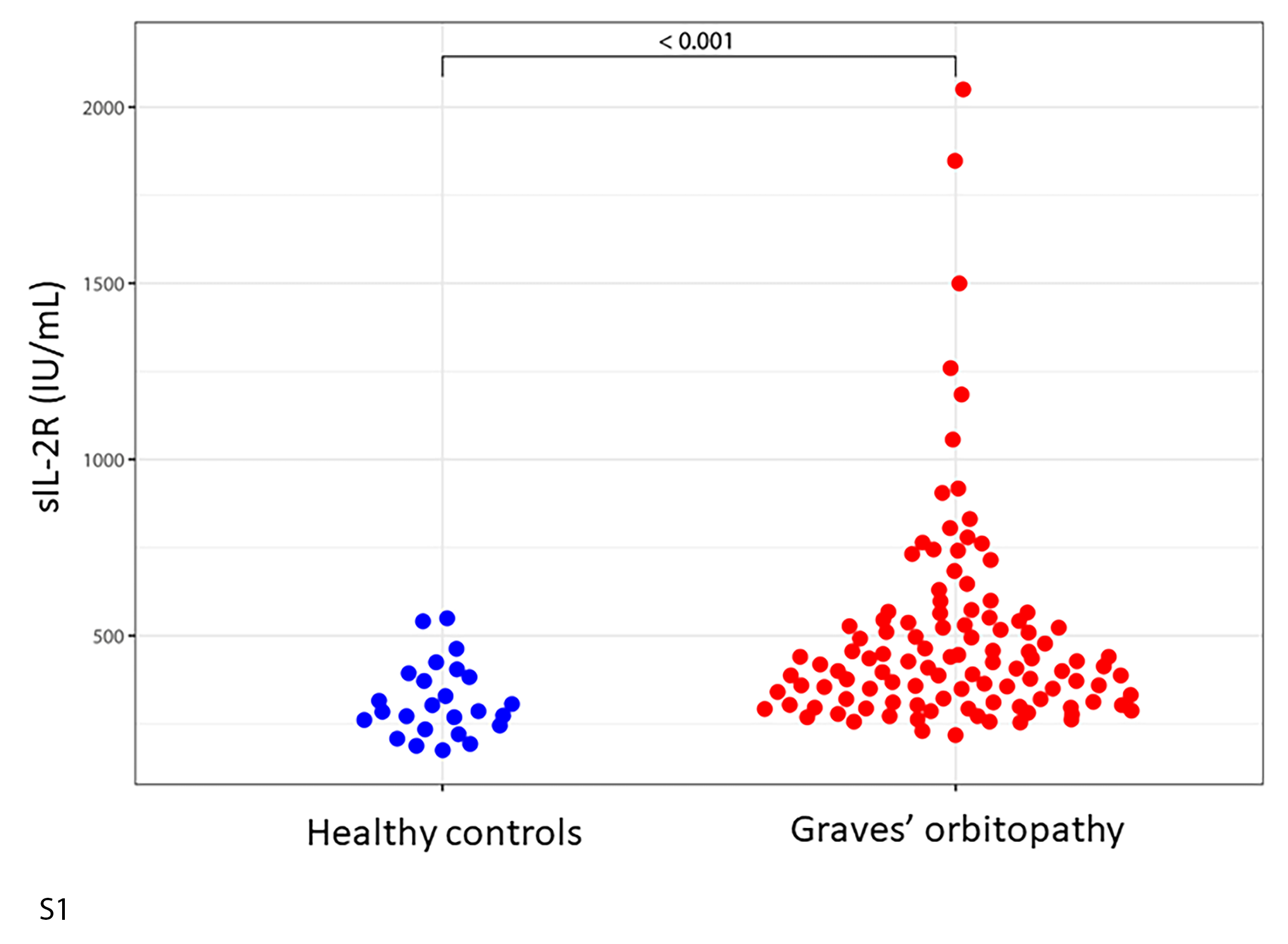

Supplement: Supplementary Figure 1 — sIL-2R levels in GO patients and healthy controls. sIL-2R levels are significantly higher in GO patients (418 IU/mL, IQR = 225) compared to healthy controls (286 IU/mL, IQR = 149; p < 0.001). [file DataSheet_1.zip › Supplementary Figure 1.TIF]

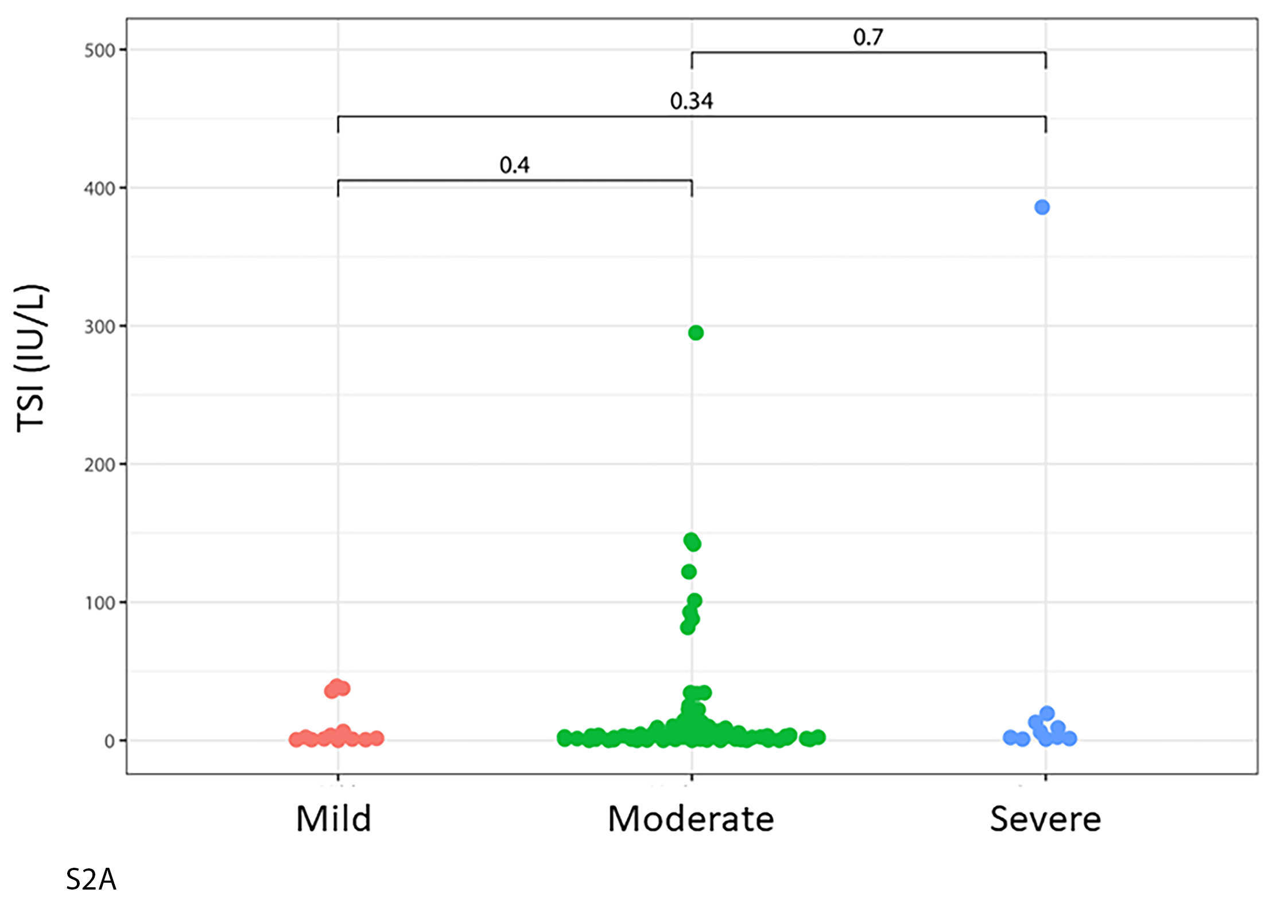

Supplement: Supplementary Figure 1 — sIL-2R levels in GO patients and healthy controls. sIL-2R levels are significantly higher in GO patients (418 IU/mL, IQR = 225) compared to healthy controls (286 IU/mL, IQR = 149; p < 0.001). [file DataSheet_1.zip › Supplementary Figure 2A.TIF]

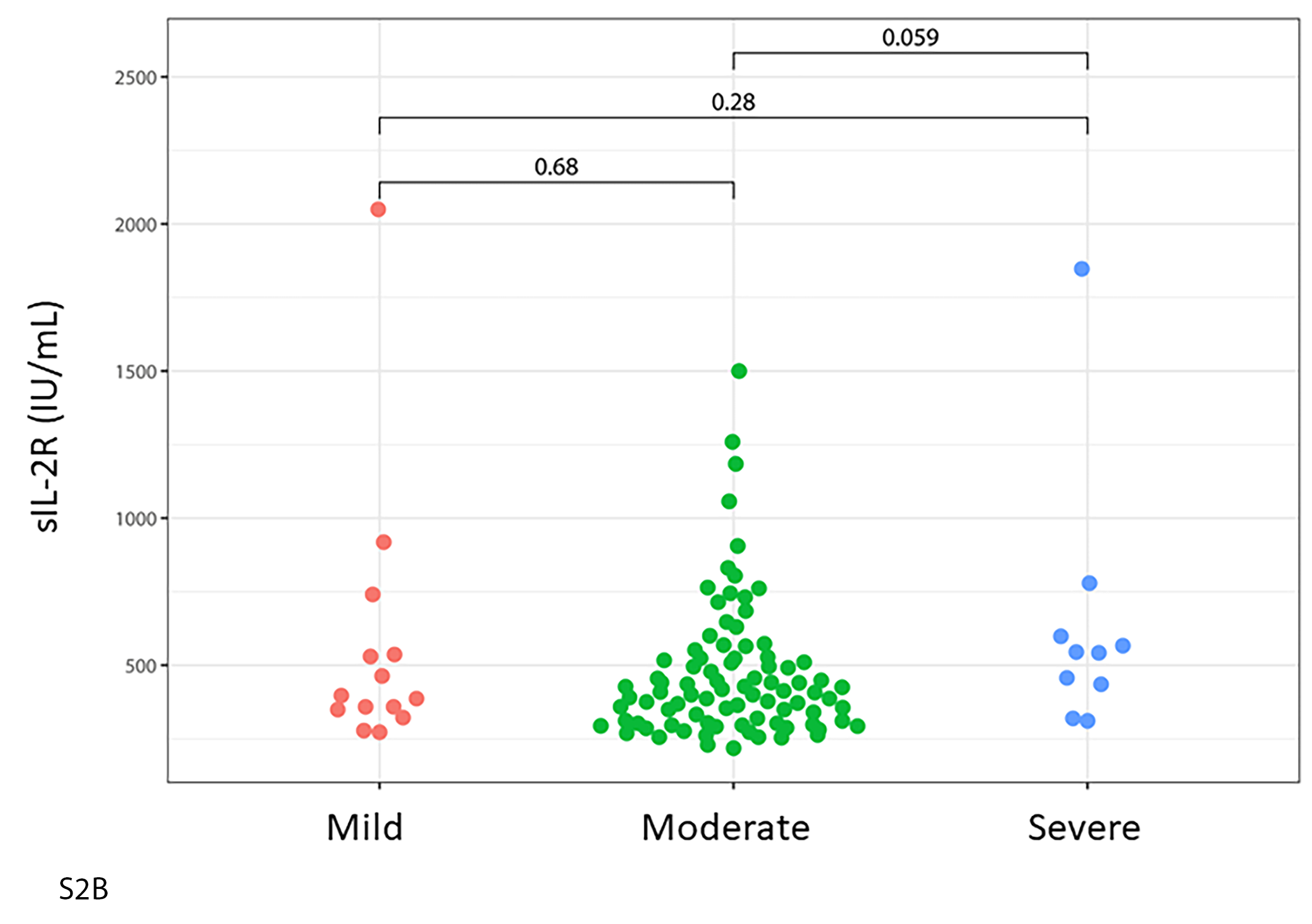

Supplement: Supplementary Figure 1 — sIL-2R levels in GO patients and healthy controls. sIL-2R levels are significantly higher in GO patients (418 IU/mL, IQR = 225) compared to healthy controls (286 IU/mL, IQR = 149; p < 0.001). [file DataSheet_1.zip › Supplementary Figure 2B.TIF]

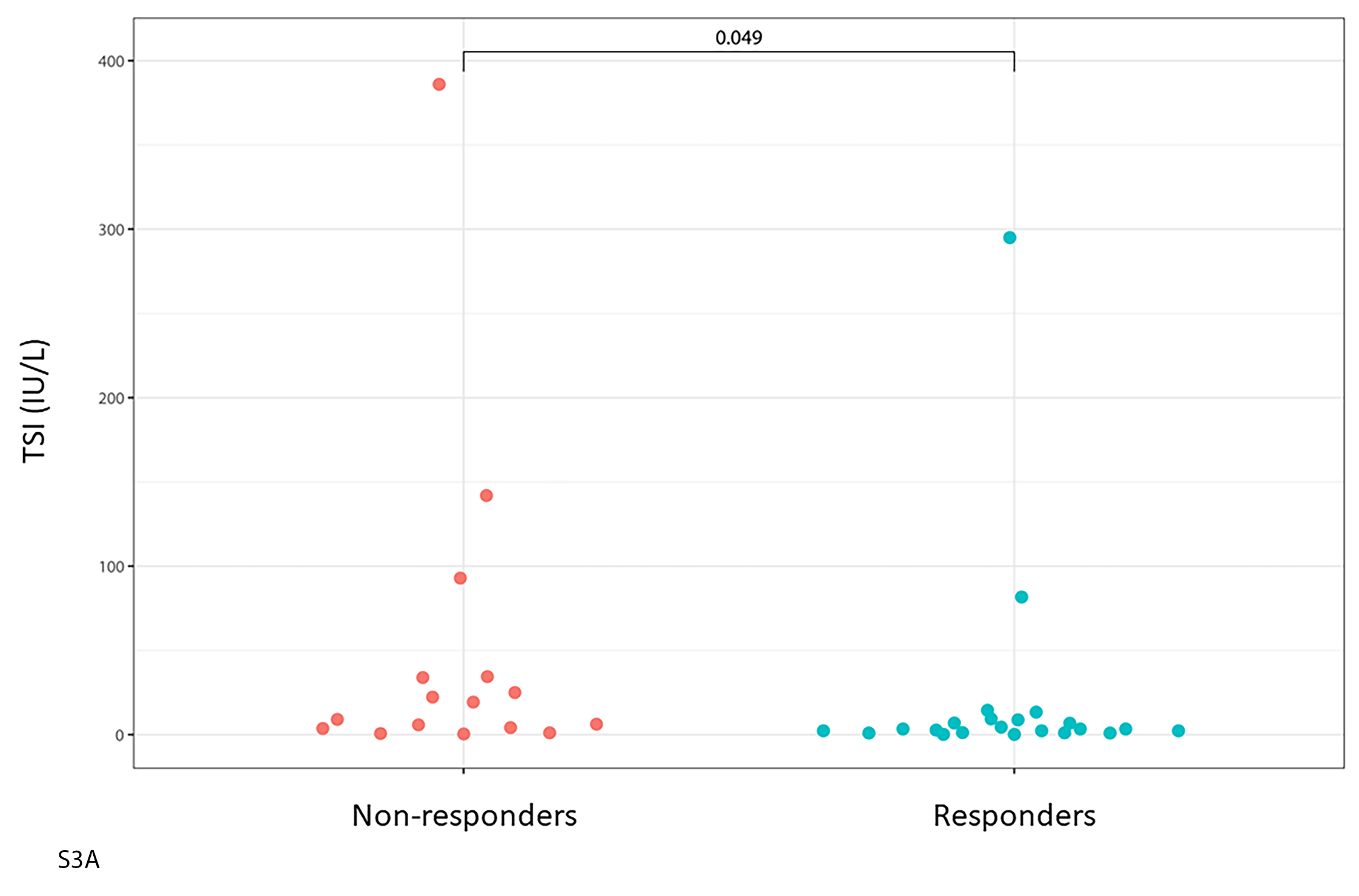

Supplement: Supplementary Figure 1 — sIL-2R levels in GO patients and healthy controls. sIL-2R levels are significantly higher in GO patients (418 IU/mL, IQR = 225) compared to healthy controls (286 IU/mL, IQR = 149; p < 0.001). [file DataSheet_1.zip › Supplementary Figure 3A.TIF]

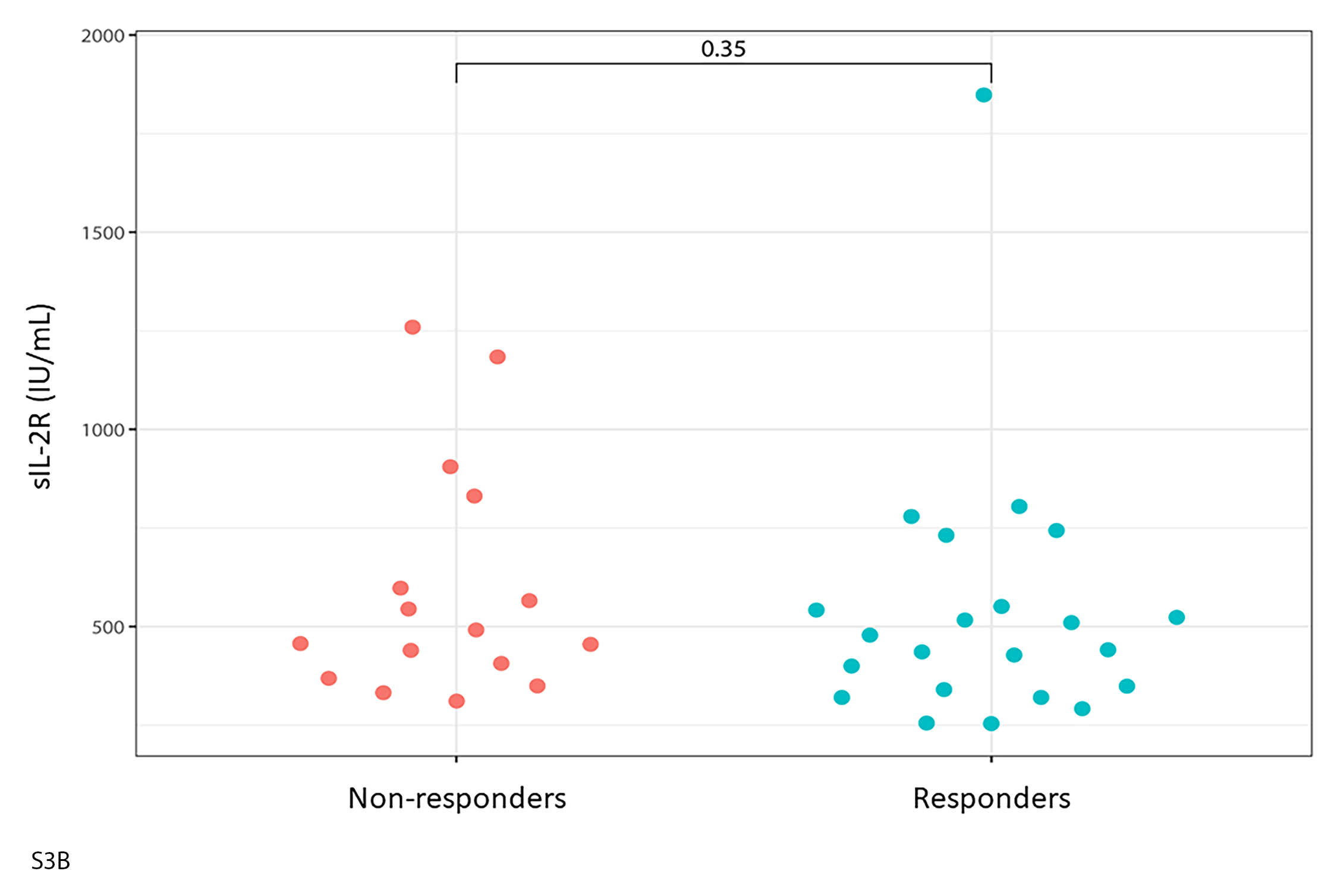

Supplement: Supplementary Figure 1 — sIL-2R levels in GO patients and healthy controls. sIL-2R levels are significantly higher in GO patients (418 IU/mL, IQR = 225) compared to healthy controls (286 IU/mL, IQR = 149; p < 0.001). [file DataSheet_1.zip › Supplementary Figure 3B.TIF]
